# Impact of the Individualized Positive Psychosocial Interaction (IPPI) ePCT on Staff Care Partner Outcomes

**DOI:** 10.1093/geroni/igaf122.316

**Published:** 2025-12-31

**Authors:** Katherine Abbott, Allison R Heid, Molly Noble, Kathleen Unroe, Amy Kotterman, Kimberly Van Haitsma

**Affiliations:** Miami University, Oxford, Ohio, United States; Independent Research Consultant, Ardmore, Pennsylvania, United States; Miami University, Oxford, Ohio, United States; Indiana University School of Medicine, Indianapolis, Indiana, United States; United Church Homes, Marion, Ohio, United States; The Pennsylvania State University, University Park, Pennsylvania, United States

## Abstract

The Individualized Positive Psychosocial Interaction (IPPI) program is an evidence-based program that engages persons living with dementia in nursing homes in brief (i.e., 10 minute) one-to-one preference-based activities two times a week. Primary direct care providers of eligible intervention recipients (i.e., CNAs, activity professionals, nurses, and social workers) are trained to provide IPPI. This study examined the impact of program training and implementation on staff care partner outcomes of knowledge, self-efficacy, appropriateness, acceptability, satisfaction, meaningfulness of time, and well-being. Seventy staff care partners completed an online course on emotion-focused communication strategies (EFCT) with a pre-and post-training assessment of knowledge and self-efficacy. Post-training appropriateness, acceptability, and satisfaction were assessed. Care partners were then trained on IPPI delivery and implemented the program 2x a week with residents for 6-months each. Immediately following each IPPI, they rated their perception of meaningfulness of time spent. Nineteen of the care partners also completed an exit interview rating the impact of the IPPI program on their well-being. Knowledge of emotion-focused communication strategies and self-efficacy for using emotion-focused communication strategies significantly increased from pre- to post-training. Acceptability, appropriateness, and satisfaction with intervention training were also high. Following 92% (n = 3,639) of IPPIs, staff care partners indicated that they found the IPPI to be a meaningful use of their time. Mean levels of well-being associated with delivering the IPPI were also high. IPPI is a person-centered, preference-based intervention that improves outcomes for both the residents receiving the intervention and the staff care partner providing the services.

